# Multi-locus phylogeography of the dusky dolphin (*Lagenorhynchus obscurus*): passive dispersal via the west-wind drift or response to prey species and climate change?

**DOI:** 10.1186/1471-2148-7-131

**Published:** 2007-08-03

**Authors:** April D Harlin-Cognato, Tim Markowitz, Bernd Würsig, Rodney L Honeycutt

**Affiliations:** 1 Department of Zoology, Michigan State University, 203 Natural Sciences Building, East Lansing, MI 48824, USA.; 2 Dusky Dolphin Research Project, Muritai, 13 Maui Street, Kaikoura, New Zealand.; 3 Marine Mammal Research Program, Department of Marine Biology, Texas A&M University, 4700 Ave. U, Galveston, Texas 77551, USA.; 4 Pepperdine University, Natural Science Division, 24255 Pacific Coast Highway, Malibu, California 90263, USA.

## Abstract

**Background:**

The dusky dolphin (*Lagenorhynchus obscurus*) is distributed along temperate, coastal regions of New Zealand, South Africa, Argentina, and Peru where it feeds on schooling anchovy, sardines, and other small fishes and squid tightly associated with temperate ocean sea surface temperatures. Previous studies have suggested that the dusky dolphin dispersed in the Southern Hemisphere eastward from Peru via a linear, temperate dispersal corridor provided by the circumpolar west-wind drift. With new mitochondrial and nuclear DNA sequence data, we propose an alternative phylogeographic history for the dusky dolphin that was structured by paleoceanographic conditions that repeatedly altered the distribution of its temperate prey species during the Plio-Pleistocene.

**Results:**

In contrast to the west-wind drift hypothesis, phylogenetic analyses support a Pacific/Indian Ocean origin, with a relatively early and continued isolation of Peru from other regions. Dispersal of the dusky dolphin into the Atlantic is correlated with the history of anchovy populations, including multiple migrations from New Zealand to South Africa. Additionally, the cooling of the Eastern Equatorial Pacific led to the divergence of anchovy populations, which in turn explains the north-south equatorial transgression of *L. obliquidens *and the subsequent divergence of *L. obscurus *in the Southern Hemisphere.

**Conclusion:**

Overall, our study fails to support the west-wind drift hypothesis. Instead, our data indicate that changes in primary productivity and related abundance of prey played a key role in shaping the phylogeography of the dusky dolphin, with periods of ocean change coincident with important events in the history of this temperate dolphin species. Moderate, short-term changes in sea surface temperatures and current systems have a powerful effect on anchovy populations; thus, it is not infeasible that repeated fluctuations in anchovy populations continue to play an important role in the history of coastal dolphin populations.

## Background

**Figure 1:**
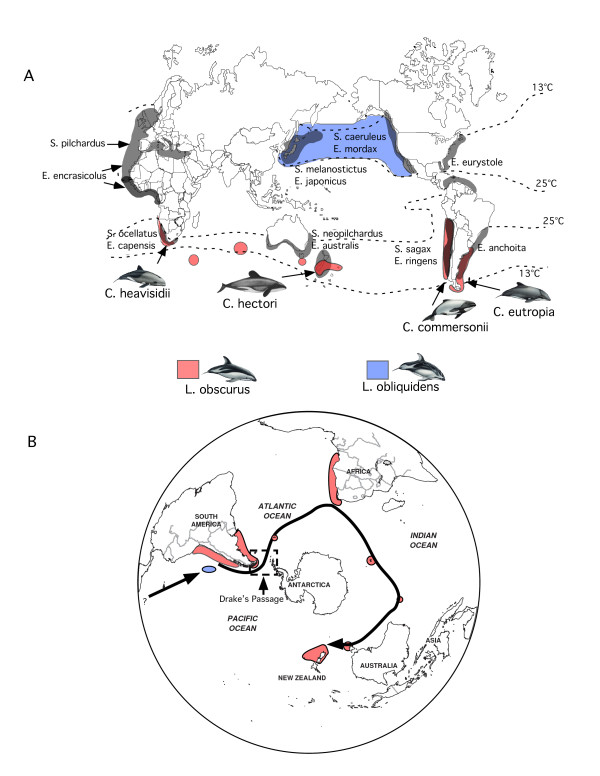
**Global distribution of the dolphin genera *Lagenorhynchus *and *Cephalorhynchus *and associated prey species in temperate ocean regions**. (A) Grey shaded areas outline the distribution of sardines (*Sardinops*, *Sardina*) and anchovy (*Engraulis*), which are regionally bounded by the 13°C and 25°C isotherms (dotted curves; modified from Grant and Bowen [50]). Coloured areas indicate the distribution of *Lagenorhynchus *species. *Cephalorhynchus *distributions overlap partially with *Lagenorhynchus *species in all areas indicated with an arrow. The distribution of dolphin species with anchovy and sardines is indicated where coloured and grey shaded areas overlap along temperate, coastal regions. (B) The west-wind drift hypothesis suggests that the dusky dolphin (*L. obscurus*) dispersed via a circum-global band of uninterrupted cool water, starting in coastal Peru, moving eastward through Drake's passage, to Argentina, South Africa, and New Zealand.

Over a 2.5 million year period in the Pleistocene repeated patterns of expansion and recession of ice sheets from Antarctica influenced globally the distribution of sea surface temperatures and the flow of ocean currents [1-3]. These historical geological events during the Plio-Pleistocene influenced contemporary patterns of genetic structure observed in marine organisms living in the Southern Hemisphere [4-6]. For example, small-bodied dolphins of the genera *Cephalorhynchus *and *Lagenorynchus *occupy temperate waters in the Southern Hemisphere and share similarities in their distribution and pattern of morphological and genetic (exclusively mtDNA) divergence that have been interpreted as being the result of dispersal corridors present during inter-glacial periods in the Plio-Pleistocene [7,8]. These contemporary patterns of divergence and distribution observed for these genera of dolphin inspired the west-wind drift hypothesis, which suggests an eastward dispersal of dolphins via a unidirectional, linear route with the temperate west-wind drift ocean current present during the Plio-Pleistocene. The west-wind drift hypothesis plus the preference for temperate waters purportedly explains the roughly concordant distribution of these dolphins in temperate, upwelling regions along coastal South Africa, South America, and New Zealand within 8°C and 16°C isotherms (Figure 1). As a result, the current ranges of these genera are large but discontinuous, separated by vast expanses of ocean and tropical waters between temperate regions.

*Lagenorhynchus obscurus *(the dusky dolphin) is the one of the most widely distributed dolphin species in the Southern Hemisphere, with a disjoint distribution restricted to temperate waters (Figure 1). As such, this species provides a model for testing several predictions explicit in the west-wind drift hypothesis. First, the west-wind drift hypothesis implies a Peruvian origin of the dusky dolphin followed by eastward dispersal to other regions (Figure 1) [7,9]. In support of this prediction molecular studies have proposed that the Peruvian population was founded when *L. obscurus *and *L. obliquidens*, an anti-tropically distributed sister-species pair [10,11], diverged during a southward equatorial transgression in the Pacific Ocean approximately 1 million years ago when tropical waters cooled [12,13]. In contrast, recent molecular studies with better geographic sampling and both nuclear and mitochondrial DNA markers indicate a phylogeographic history of the dusky dolphin *inconsistent *with the west-wind drift hypothesis [8,14]. These studies suggest an alternative Atlantic/Indian Ocean origin [8], which is inconsistent with an anti-tropical speciation event in the Pacific Ocean and necessitates an alternative explanation for the transition between oceans.

Second, dispersal via the west-wind drift should result in a pattern of genetic variation that is linear and unidirectional [8,15]. One molecular study [14] suggests that Argentina, South Africa, and New Zealand populations have experienced relatively recent contact, perhaps as a large ancestral Atlantic/Indian Ocean population. Therefore, the origin of the dusky dolphin and its phylogeographic history remain inconclusive.

Finally, west-wind drift represents a passive dispersal mechanism that would result in a distributional pattern of the dusky dolphin that should be independent of changes in sea-surface temperatures, prey distribution, and correlated primary productivity. The dusky dolphin is concentrated in regions of high primary productivity, where cyclic upwelling supports an abundance of schooling anchovy (*Engraulis *sp.), sardines (*Sardinops *sp.), and other small fishes and squid limited in their distribution to temperate regions (Figure 1). The geographic association of these fishes and the dusky dolphin is not coincidental. Stomach-content analysis and behavioral observations in Argentina, Peru, and New Zealand indicate the diet of the dusky dolphin is comprised mainly of these prey items [13,16], and that movements of dolphin populations are coincident with seasonal shifts in prey distribution [17,18]. The preference for temperate prey species is an ancestral characteristic of all members of the genus *Lagenorhynchus*, including the Pacific white-sided dolphin (*L. obliquidens*), whose distribution is correlated with that of North Pacific anchovy (Figure 1). Sardines and anchovy are regionally and physiologically bounded by 13°C and 25°C isotherms (Figure 1) [4]. Clearly, the phylogeographic history of the dusky dolphin and its temperate prey species are biologically linked, so that global climatic oscillations affecting temperate regions would produce a history correlated with the isolation of ocean basins, with the degree of regional genetic divergence concordant with the closure of temperate ocean corridors and the frequency and duration of fluctuations in sea surface temperatures that altered the distribution and availability of prey.

In this study, we use new nuclear and mitochondrial DNA sequence data and complete geographic sampling to challenge the role that the west-wind drift played in the genetic structuring and dispersal of dusky dolphin populations. We provide a global phylogeographic perspective of the origin of the dusky dolphin, and assess the historical patterns of gene flow related to the location of temperate, up-welling coastal regions that are tightly linked to the distribution of prey species. In contrast to previous studies, our data support an alternative to the west-wind drift hypothesis, with a temporal and environmental correlation between the phylogeographic history of the dusky dolphin, its anchovy prey, and the Plio-Pleistocene paleoceanography of the Southern Ocean and Eastern Equatorial Pacific.

## Results

### Diversity indices

Table 1 summarizes indices of genetic diversity. With the exception of cyt *b *for Peru, haplotype and nucleotide diversity for the cyt *b *and control region was high. The lower diversity of cyt *b *in Peru was first described by Cassens et al. [14] and was attributed to high levels of mortality along the Peruvian coastline.

**Table 1:** Summary of genetic diversity indices for cytochrome *b*, control region, and actin intron I sequence data

Partition	Region	n	*h*	π	S
cytochrome *b*	AR	31	0.93 (± 0.030)	0.0046 (± 0.003)	32
	SA	37	0.91 (± 0.027)	0.0047 (± 0.003)	34
	NZ	23	0.91 (± 0.052)	0.0063 (± 0.003)	37
	PE	78	0.71 (± 0.048)	0.0024 (± 0.001)	18
		
	Total	169			

control region	AR	29	0.91 (± 0.048)	0.0066 (± 0.004)	10
	SA	89	0.99 (± 0.005)	0.0170 (± 0.009)	47
	NZ	252	0.98 (± 0.005)	0.0187 (± 0.010)	82
	PE*	118	0.95 (± 0.010)	0.0123 (± 0.007)	N/A
		
	Total	488			

actin	AR	14	0.70 (± 0.095)	-	4
	SA	44	0.76 (± 0.049)	-	7
	NZ	48	0.82 (± 0.028)	-	7
		
	Total	106		-	

*From Cassens et al. [14]; AR = Argentina, SA = South Africa, NZ = New Zealand, PE = Peru. n = number of mitochondria or gametes examined. *h *= haplotypic diversity, π = nucleotide diversity. S = number of segregating sites for mtDNA regions, or the number of actin alleles present in each geographic region. Standard errors are in parentheses.

### Prediction 1: Peruvian origin

**Figure 2:**
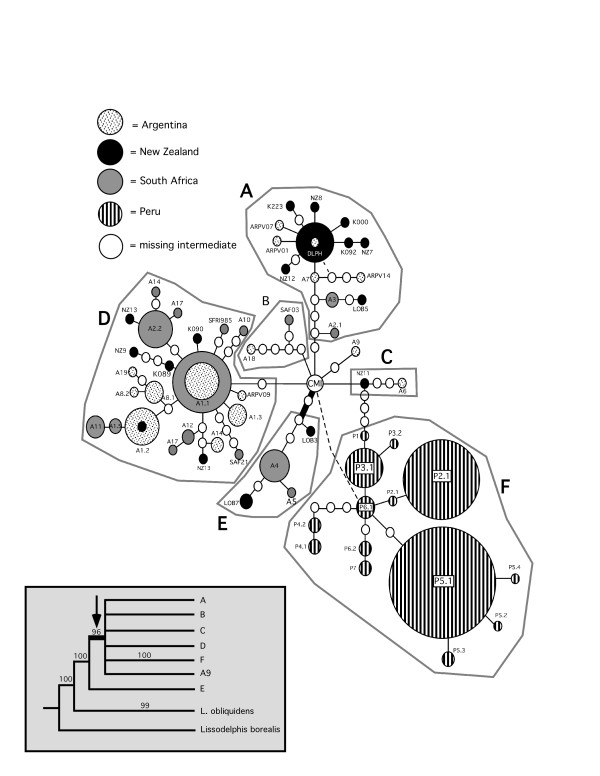
**The statistical parsimony network of dusky dolphin cytochrome *b *haplotypes**. Gray polygons indicate haplogroups with a Bayesian posterior probability of 90% or greater. The thick branch indicates the probable root of the dusky dolphin phylogeny, and dotted lines indicate alternative connections (reticulations) between haplotypes. Note the lack of a central haplotype, the "CMI" as in Cassens et al. [8]. Boxed insert is a condensed version of the Bayesian analysis consensus tree, with letters corresponding to haplogroups in the nested network. As in the haplotype network, the thick branch under the arrow is the proposed root of dusky dolphin lineages with numbers above nodes indicating posterior probabilities derived from Bayesian analysis.

**Figure 3:**
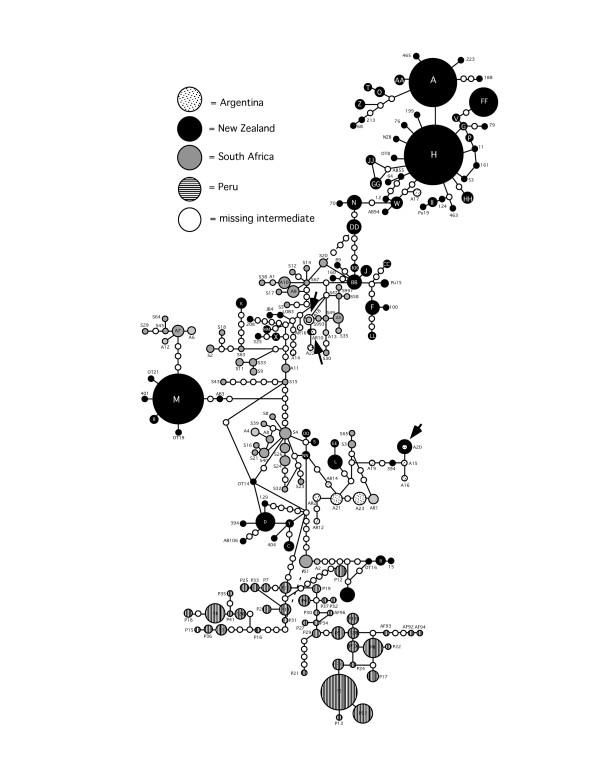
**The statistical parsimony network of dusky dolphin control region (d-loop) haplotypes**. Arrows indicate haplotypes sampled in more than one geographic region. Dotted lines indicate alternative connections (reticulations) between haplotypes.

The dusky dolphin d-loop and cyt *b *haplotype networks recovered a Peru-specific lineage divergent from other geographic regions by several missing intermediate haplotypes (Figures 2 and 3). Similarly, the Bayesian analysis recovered a monophyletic clade of Peruvian haplotypes with 100% posterior probability, and generally supported the phylogenetic pattern of the statistical parsimony networks (Figure 2). The Bayesian analysis indicated with 96% posterior probability that the root of the dusky dolphin phylogeny was not the Peruvian clade, but rather occurred along a lineage of South American and New Zealand haplotypes (Figure 2).

### Prediction 2: Linear dispersal route

For each of the mitochondrial and nuclear genetic markers, both an AMOVA and F_st _values revealed significant partitioning of variation among Peru, Argentina, New Zealand, and South Africa (Table 2). The high levels of divergence reflect the very low number of haplotypes found in more than one geographical region. For example, only 3 cyt *b *(A1.2, A1.1, and DLPH; Figure 2) and d-loop (A20, AR10, A18; Figure 3) haplotypes occurred in >1 geographic region. In no instance were Peruvian mitochondrial haplotypes shared with any other locality. Thus, the highest F_st _values were between Peru and other localities, with the level of divergence between South Africa and Argentina considerably lower than either was to New Zealand (Table 2). Despite the high degree of divergence between geographic regions, no apparent pattern of a linear, easterly increase in divergence was evident.

**Table 2:** Genetic divergence among *L. obscurus *populations.

**A. AMOVA**			**B. Pairwise divergence**					
	Source	% variation	φ_st_					
**Cytb**					NZ	AR	SA	PE

(NZ, AR, SA, PE)	Among populations	48.95		NZ	-			
			0.49**	AR	0.21**	-		
	Within Populations			SA	0.29**	0.05*	-	
		51.05		PE	0.61**	0.59**	0.59**	-

**Dloop**								

(NZ, AR, SA, PE)	Among populations	73.90		NZ	-			
			0.74**	AR	0.83**	-		
	Within populations	26.10		SA	0.85**	0.08**	-	
				PE	0.47**	0.74**	0.78**	-

**Actin**								

(NZ, AR, SA)	Among populations	22.59		NZ	-			
			0.23**	AR	0.23**	-		
	Within populations	77.41		SA	0.21**	0.26**	-	

A) Analysis of molecular variance for cyt *b*, control region, and actin intron sequence data. B) Pair-wise F_st _values. NZ = New Zealand, AR = Argentina, SA = South Africa, PE = Peru. * = P < 0.01; ** = P < 0.001

**Figure 4:**
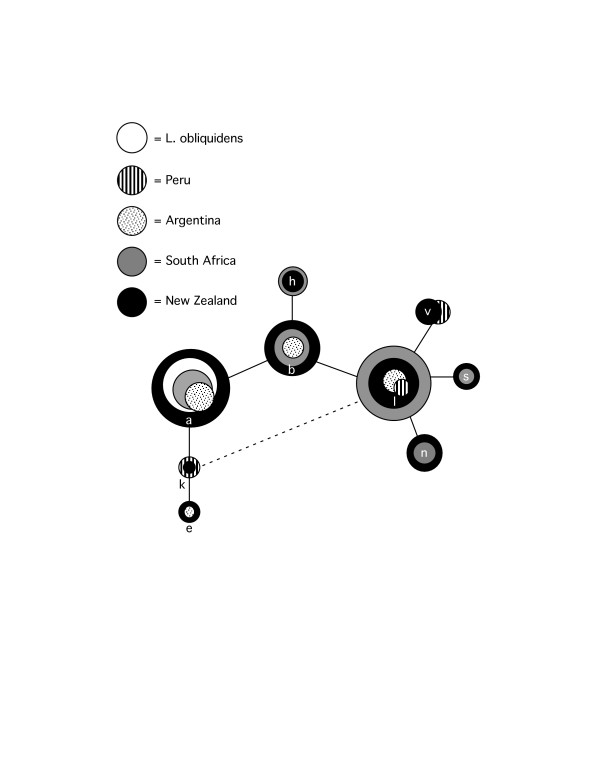
**The statistical parsimony network of dusky dolphin actin intron I alleles**. Dotted lines indicate alternative connections (reticulations) between haplotypes.

The geographic distribution of haplotypes within the statistical parsimony networks revealed a genetic signature inconsistent with a linear dispersal route. In both cyt *b *(Figure 2) and control region (Figure 3) networks, New Zealand lineages were notably separated from the Peruvian clade by a mixture of haplotypes from South Africa, Argentina, New Zealand, and several missing haplotypes. With the exception of Peru, at least one haplotype at the distal tip of each central haplotype group was from New Zealand. Both mitochondrial networks lacked a central haplotype in high frequency, with the majority of most frequent haplotypes clustered at the tips of the networks. The actin network (Figure 4) was dominated by three common alleles (a, b, l) at the center of the network present. These alleles varied in frequency in all four biogeographic regions and in the sister taxon, *L. obliquidens*. In 3 of the 5 tip clades, alleles were common to South Africa and New Zealand. The tips of the network contained alleles present in lower frequency and with more restricted geographic distributions. In one instance an allele from Argentina was placed at the periphery of the network (haplotype 'e'), and this allele was also sampled in New Zealand.

**Figure 5:**
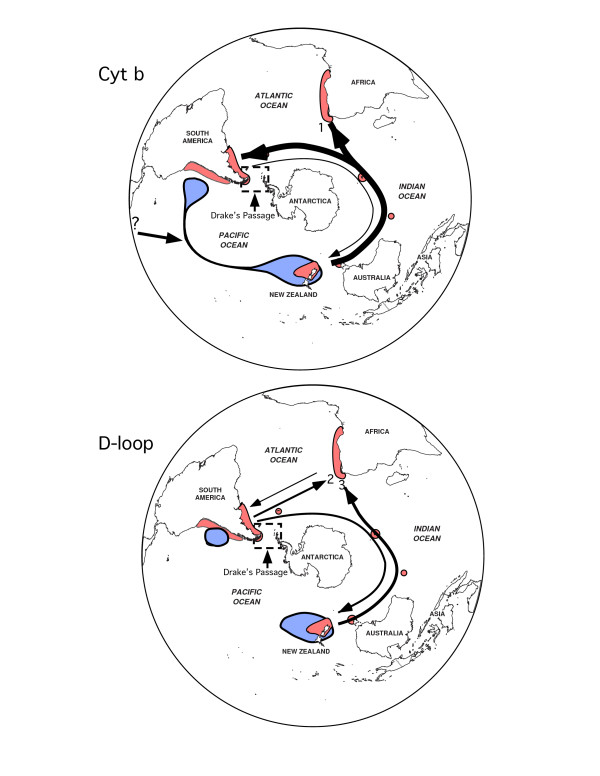
**Dispersal hypothesis synthesized from analyses of cytochrome *b *and d-loop sequence data**. Blue shaded areas depict the hypothesized origin of the dusky dolphin from a trans-equatorial transgression of *L. obliquidens*, approximately 2 million years ago, that resulted in an early isolation of a Peruvian population and the founding of a population in New Zealand, which served as the source population for migration into the Atlantic. Arrow direction and thickness indicate the direction and magnitude (Table 3) of migrations, respectively. Only migration rates ≥ 1, the theoretical minimum necessary to maintain population cohesion, are depicted (Table 3). Under the assumption that the rate of evolution of the cyt *b *and d-loop regions vary, the comparison of the cyt *b *with the d-loop allows for a relative temporal interpretation of migration rates, with those from the d-loop representing more recent patterns. Based on this assumption, numbers at the end of arrows indicate the proposed 2–3 migrations into South Africa, perhaps following extirpation due to climatic fluctuations (see Discussion).

The MIGRATE analysis produced estimates of the amount of migration between geographical regions that suggests multiple east to west migrations dominated by dispersal from New Zealand to South Africa and Argentina and an absence of migration from Peru to other regions (Figure 5). The relative temporal shift in migration rates is evident in the comparison of the independent analyses of cyt *b *and the d-loop. The analysis of the cyt *b *gene suggests early high levels of migration from New Zealand to South Africa and Argentina with weak or no migration from South Africa or Argentina to Peru. The d-loop suggests a reduction in the rate of migration from New Zealand to South Africa and Argentina when compared to cyt *b*. In the estimates from the d-loop a weak, but significant, migration is observed from Argentina to South Africa and New Zealand. This was not represented in the cyt *b *results. Overall, these results support: 1) early and continued isolation of Peru from other regions; 2) at least 1, and possibly 2, periods of migration from New Zealand to South Africa, with the first migration period represented by cyt *b *suggesting a high, unidirectional migration rate; 3) the later onset of migration from Argentina to South Africa representing a possible 3^rd ^period of migration into South Africa; 4) a high rate of unidirectional migration from New Zealand to Argentina correlated with that of migration to South Africa, suggesting an eastward dispersal event from New Zealand, and 5) followed by a reduced migration rate between New Zealand, Argentina, and South Africa, as would be expected of populations that gradually became genetically isolated.

### Prediction 3: Dates of divergence

Divergence times calculated from cyt *b *data indicated a divergence between *L. obscurus *and *L. obliquidens *of 1.9 [95% CI: 1.3–2.9] million years ago (mya), and the Peruvian lineage separated from other geographic regions 0.63 [95% CI: 0.38–0.98] mya.

### Comparative phylogeography of anchovy

The Bayesian analysis recovered a phylogeny (not shown) that supported previously established relationships between New World species of *Engraulis *with two major lineages, a "New World" clade with California (*E. mordax*) and a monophyletic South American (*E. ringens*/*E. anchoita*) lineage in which *E. ringens *and *E. anchoita *are reciprocally monophyletic, and an "Old World" clade comprised of a polyphyletic assemblage of *E. australis*, *E. encrasicolus*, *E. capensis*, and *E. japonicus *[6,50]. Our results suggest a divergence between *E. mordax *and South American species at 9.0 [95% CI: 6.9–10.0] mya, with *E. ringens *and *E. anchoita *diverging at 1.6 [95% CI: 1.0–2.5] mya.

**Figure 6:**
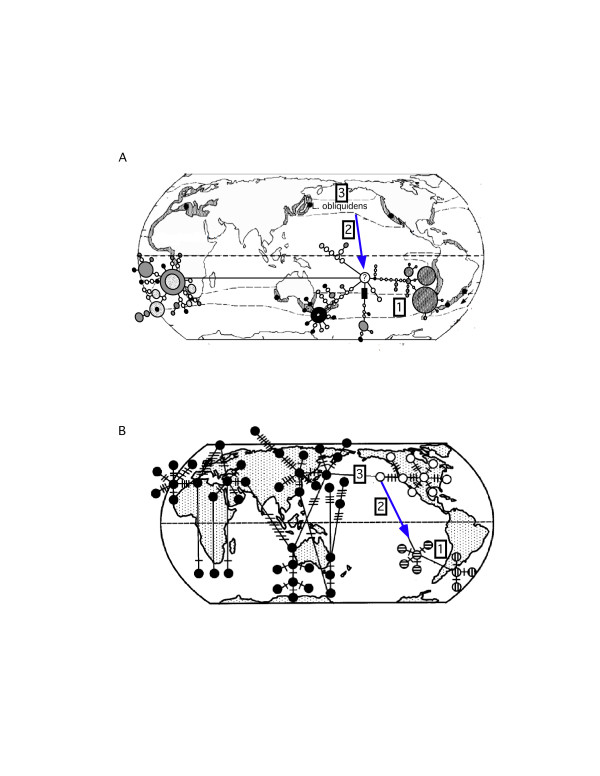
**A comparison between the global distribution of (A) dusky dolphin and (B) anchovy (*Engraulis *sp.) genetic variation**. The statistical parsimony networks of the dusky dolphin and anchovy cytochrome *b *haplotypes are overlaid on a map to illustrate the geographic pattern of haplotype evolution. Numbers point out notable similarities: (1) marked divergence between South American haplotypes from those in the Atlantic and Indian Oceans, and (2) north-south equatorial transgressions in the Pacific Ocean with (3) a putative ancestor in the North Pacific. Anchovy illustration is from Grant and Bowen [50].

The parsimony network of anchovy cyt *b *sequences displayed similar geographical patterns in the distribution of genetic diversity as that of the dusky dolphin (Figure 6).

## Discussion

### Dusky dolphin phylogeography and the west-wind drift hypothesis

**Figure 7:**
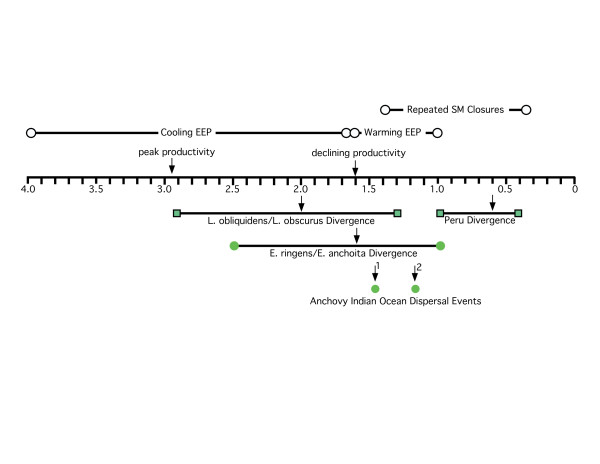
**A chronological correlation of paleoceanographic events and the phylogeographic histories of the dusky dolphin and anchovy (*Engraulis *sp)**. Above the timeline are paleoceanographic events, with the point estimates and range of dates for each event. EEP = Eastern Equatorial Pacific; SM = Strait of Magellan. Below the timeline are events in the history of anchovy and dusky dolphin populations. Bars represent 95% confidence intervals for point estimates, indicated by black arrows. At least as early as 4.3 mya, a cooling trend began in the Eastern Equatorial Pacific (EEP), causing sea surface temperatures in this tropical region to drop from 26°C to as low as 20°C in the late Pleistocene [51]. This drop in temperature caused a peak in primary productivity 2.9 mya that was 2 to 3 times greater than average values earlier in the Plio-Pleistocene [51]. At 1.6 mya, this productivity began to decline as sea surface temperatures began to rise [51]. As warmer tropical waters returned, the range and abundance of anchovy in the EEP would have declined, resulting in the gradual disappearance of this dispersal corridor, consequently resulting in an anti-tropical division of dolphin populations.

The overall pattern of genetic variation observed for the dusky dolphin in the Southern Hemisphere is not congruent with the west-wind drift hypothesis. Bayesian analysis of mitochondrial DNA sequences suggests the dusky dolphin diverged from *L. obliquidens *via a north to south equatorial transgression approximately 2 million years ago, with confidence intervals overlapping previously published estimates. However, the root of the dusky dolphin dispersal in the Southern Hemisphere was not Peru as previously proposed [13,19], but rather somewhere in the southern South Pacific/Indian Ocean basins. This result is further supported by the divergence of Peru from other regional populations relatively recently, and considerably later than the *L. obliquidens*/*L. obscurus *divergence 2.0 million years ago (Figure 7). There are several lines of evidence from the distribution of genetic variation that support a larger, South Pacific/Atlantic Ocean population as the source of dusky dolphin lineages. First, the presence of haplotypes from New Zealand at both the center and the periphery of the actin network suggest an old New Zealand population. Second, the central cluster of haplotypes from New Zealand, South Africa, and Argentina in low frequencies at the center of the mitochondrial DNA haplotype networks (Figures 2 and 3) is indicative of the remnants of a historical population with a large geographical distribution that did not include Peru. Third, there are greater levels of genetic divergence between New Zealand and other regions, and the lowest levels of divergence between South Africa and Argentina (Table 2). This pattern of genetic divergence is consistent with an early transgression to the South Pacific/Indian Ocean followed by expansion into the Atlantic.

Given that Peru is not the probable root of dusky dolphin origin, there are two alternative scenarios to explain the marked isolation of Peru from other geographical regions, both of which support a South Pacific/Indian Ocean origin. First, the north to south transgression during the Plio-Pleistocene resulted in the formation of a large population of dusky dolphins that eventually extended from New Zealand to Australia, South Africa, Argentina, and dispersed through Drake's passage between the Antarctic Peninsula and Tierra del Fuego into Peru. There is evidence that glacial cycles had profound effects on the availability of a temperate dispersal corridor for the dusky dolphin from Peru into the Atlantic, both through the Strait of Magellan and Drake's Passage [20-23]. These alterations in the availability of a dispersal route may have resulted in the isolation of a Peruvian population from other regions, and may explain the contemporary gap in the distribution of the dusky dolphin along the Peruvian coast from the Strait of Magellan to southern Tierra del Fuego (Figure 1). Second, equatorial transgression led to a large, cohesive population of dusky dolphins that extended from Peru across the southern Pacific and Indian Oceans. Changes in migration patterns across the southern Pacific, coupled with restricted dispersal through Drake's passage and the Strait of Magellan resulted in the early isolation of Peru from other geographic regions. Although our data are insufficient to discriminate between these alternative hypotheses, neither is consistent with a Peruvian origin.

Our analysis of the phylogeography of the dusky dolphin does not support a linear, eastward dispersal route predicted by the west-wind drift hypothesis. Our data do not show a pattern of isolation-by-distance from Peru to New Zealand. To the contrary, historical migration patterns suggest an alternative hypothesis of a founding population in the South Pacific/Indian Ocean that expanded its geographic range to the Atlantic via a temperate dispersal corridor (Figure 5). Evidence from analysis of cyt *b *and d-loop sequences suggests that the migration rate per generation from New Zealand westward was highest relatively early, followed by continued, but reduced, westward migration later in history. This reduction in westward migration was accompanied by weak, but potentially significant, migration from Argentina eastward to South Africa and New Zealand (Table 3, Figure 5). This hypothesis receives further support from the occurrence of very few haplotypes in more than one geographic region, and the relatively large number of missing intermediates between haplotypes typical of an old population subject to the effects of genetic drift. In the Southern Hemisphere, eastern South America, South Africa, and New Zealand are loosely connected by a series of small islands within a band of temperate sea surface temperatures (Figure 1). These islands are used at least periodically by the dusky dolphin [9,24] and likely served as stepping-stones for reciprocal genetic exchange across the South Pacific and Indian Oceans. Although the dusky dolphin may have at one time dispersed via oceanic islands, a recent study of microsatellite variation between South Africa and Argentina found no evidence for contemporary dispersal of dusky dolphins between these regions [14]. The large cluster of New Zealand-specific haplotypes at the periphery of the cyt *b *and control region networks (Figures 2 and 3), with large, mixed clusters of Argentina/South Africa haplotypes is indicative of the slow decay of contact between geographical regions over time with increased genetic distance. Overall, this suggests that the observed pattern of genetic diversity in contemporary dusky dolphin populations is the relic of a historically large population that has since become isolated into 4 regional populations.

**Table 3:** Summary of estimated migration rates per generation for dusky dolphin populations.

Population	Locus	Theta [N_e_mu]	Migrations per generation [m/mu] * theta			
			1,+	2,+	3,+	4,+

1 New Zealand	Dloop	0.009	-------	0.000	2.240	0.125
	Cytb	0.014	-------	0.282	1.477	0.000
2 Peru	Dloop	0.006	0.000	-------	0.125	0.000
	Cytb	0.015	0.000	-------	0.000	0.000
3 Argentina	Dloop	0.001	0.576	0.082	-------	0.987
	Cytb	0.002	8.639	0.000	-------	0.497
4 South Africa	Dloop	0.014	2.970	0.000	2.381	-------
	Cytb	0.012	6.946	0.000	0.000	-------

u = mutations per site per generation; m = number of migrants; N_e _= effective population size [52]. A plus sign (+) indicates the receiving population.

### Comparative phylogeography of the dusky dolphin and anchovy

The parsimony network of anchovy cyt *b *sequences displayed similar geographical patterns in the distribution of genetic diversity as that of the dusky dolphin (Figure 6). Most notable similarities were: (1) marked divergence between South American haplotypes from those in the Atlantic and Indian Oceans, and (2) north-south equatorial transgressions in the Pacific Ocean with (3) a putative ancestor in the North Pacific. The correlation between the patterns of divergence between the dusky dolphin and anchovy species are likely related to paleoceanographic conditions during the Pleistocene (Figure 7). In particular, the date of *L. obliquidens*/*L. obscurus *divergence is correlated with the cooling and subsequent warming of sea surface temperatures in the EEP, with the point estimate of equatorial transgression occurring toward the end of the cooling period when primary productivity was well beyond its peak (Figure 7). The extension of nutrient rich waters in the EEP likely offered an arena for the expansion of anchovy in the EEP northward towards the equator into the tropics, and coincidentally, the cooling of tropical waters, combined with increased prey distribution and abundance, may have provided a natural conduit for the transgression of *L. obliquidens *from the Atlantic into the Pacific. The warming of sea surface temperatures and declining productivity in the EEP coincides not only with the divergence of *L. obliquidens*/*L. obscurus*, but also with the divergence of *E. ringens *and *E. anchoita *(Figure 7). This suggests the warming of the EEP and subsequent decline in productivity narrowed the distribution of *E. ringens*, which isolated eastern and western South American populations, and consequently resulted in an anti-tropical division of dolphin populations. Based on our study, this founder dolphin population took refuge in the cooler South Pacific/Indian Ocean.

Approximately 1 million years after the equatorial transgression into the Southern Hemisphere, there is evidence for a relatively early founding of *L. obscurus *in New Zealand followed by migration westward to Argentina and South Africa (Table 3, Figure 5). In addition to the evidence from the distribution of genetic diversity, these migration events support a diffusion of the dusky dolphin through the Indian Ocean into the Atlantic, followed by colonization and expansion of populations in coastal upwelling regions. There are several inferences from the history of anchovy [6] that are correlated with the phylogeographic pattern observed in the dusky dolphin. First, anchovy species are extremely sensitive to variation in sea surface temperatures and current systems, and this has been especially true in South Africa and Australia where climate change has had a very dynamic history [6]. As a result, South African and Australian species have suffered multiple extinctions, bottlenecks, and large-scale introgressions from other regions during Plio-Pleistocene climate change. The most recent colonization of Australia was most likely via equatorial transgression from Japan. Second, there is evidence for two Pleistocene dispersals across the Indian Ocean, one at 1.5 and the other 1.2 million years ago, between Europe (Atlantic) and Japan (west Pacific) via Australia and South Africa (Atlantic/Indian Oceans) "stepping stones" [6]. Similar to anchovy, our data suggest that South Africa experienced at least 2, if not 3, independent dusky dolphin colonization events. If we assume that the cyt *b *and d-loop provide some insight into the chronology of dusky dolphin dispersal events, our data suggest dusky dolphin migration events from New Zealand and Argentina to South Africa (Figure 5) potentially coincide with the two anchovy dispersal events. The first was from New Zealand (Figure 5), followed by a second weaker migration event from Argentina and perhaps a third from New Zealand (Figure 5). These colonization events are further supported by a lack of, or weak, migration to or from South Africa during multiple periods (Table 3, Figure 5), suggesting that dusky dolphins, similar to anchovy, were extirpated from South Africa perhaps due to similar response to environmental change.

## Conclusion

In summary, the phylogeographic history of the dusky dolphin and its anchovy prey are correlated, suggesting that passive dispersal via the west-wind drift did not play a role in the history of the dusky dolphin. Instead, our data support the idea that changes in primary productivity and related abundance of prey played a key role in shaping the phylogeography of the dusky dolphin, with timing of oceanic change coincident with the dates of important events in the history of dolphin populations. Given the wide-distribution and relatively recent contact between geographic regions, it is likely that the influence of paleoceanography of the Southern Hemisphere continued until recently, or perhaps continues, to play a role in the dispersal behavior of the dusky dolphin. The dynamic fluctuations in anchovy abundance on the order of decades demonstrate how moderate, short-term change in sea surface temperatures and current systems, such as during El Nino years, are powerful enough to have dramatic effects relative to anchovy populations [6]. Dolphins have the advantage of active dispersal, and are perhaps more flexible in to short-term changes in ocean conditions than anchovy with planktonic larva dispersal. Yet in Peru, the genetic variation is considerably lower than in other geographic regions. It may be that this is due to an historical founder effect followed by long-term isolation. However, it may be that population crashes in anchovy and other temperate fishes during el Nino events, along with dolphin harvest practices, are responsible for low levels of genetic diversity in Peru. On the scale of millennia, it is not infeasible that repeated fluctuations in the abundance and distribution of anchovy and other small schooling fishes in other regions have played an important role in the history of coastal dolphin populations.

## Methods

### Data collection

DNA sequence data were derived from tissues of *L. obscurus *obtained from either skin swabs of living animals or post-mortem biopsies of beach-cast animals, or from GenBank accessions (Table 4). For tissue samples, genomic DNA was isolated with either a standard phenol-chloroform techniques or with a DNeasy Kit (Qiagen, Valencia, CA). DNA from the same individual was used to amplify and sequence all gene regions when possible. Tissues from Peru were not available, but sequences were obtained from GenBank accessions (Table 4). The complete mitochondrial cytochrome *b *(cyt *b*) gene (1040 nucleotides), 474 base pairs (bp) of the mitochondrial control region, and 995 bp of nuclear DNA (nDNA) intron I of the muscle actin gene (actin) were amplified with PCR. External primer sets included: (1) cyt *b*-5'-GAAAAACCAYCGTTGTWATTCAACT-3' and 5'GTTTAATTAGAATYTYAGCTTTGGG3'; (2) control region – tPro and Dlp5 [25]; (3) Actin- 5'-GATTTGGTCCCTCTATGTCTCT-3' and 5'-TACTTTTGAACTTGCCACCTAC-3'. PCR reactions included the following: 5 μl of PCR buffer, 25 mM MgCl, 10 mM dNTP's (2.5 mM each), 1 μl 10 mg/ml bovine serum albumin (BSA), 1 μl 10 uM primer, and 5 U of *Taq*. Conditions of the PCR were 94°C, 2 min followed by 35 cycles at 92°C, 30 s, annealing 30 s, and extension at 72°C, 30 s. Published annealing temperatures were used except: 765F/766R, 50°C; LagActin1/2, 58°C. PCR products were purified with QIAquick PCR purification kits (Qiagen, Valencia, CA). Amplicons were sequenced with ABI (Applied Biosystems, Foster City, CA) BigDye Terminator chemistry, and products were run on an ABI 377. Internal primers cyt *b *(560, 5'-GCAACCCTAACACGATTCTTCG-3'; 610, 5'-CCAGTTTCGTGTAGGAATAATAGG-3') and actin (Act5-L, 5'-CCACTACTTTAGGCAG-3'; M13Act5R-H, 5'-TGTAAAACGACGGCCAGTCTGCCTAAACTAGTGG-3' were used to sequence fragments to resolve ambiguities.

**Table 4:** A summary of the sample size and source of genetic sequence data used in this study

Species	Geographic Region	DNA Region	n This study	n GenBank	n Total	GenBank Accessions
		Cyt b				
*L. obscurus*	New Zealand		15	8	23	EF093048–EF093055*
	South Africa		10	27	37	AY257141–AY257145#
						AY257131–AY257134#
						AY257130#
	Argentina		6	25	31	EF093042–EF093047*
						AY257126–AY257129#
						AY257146–AY257147#
						AY257136–AY257140#
	Peru		0	78	78	AY257148–AY257161#
		**TOTAL**	31	138	**169**	
	
		D-loop				
	New Zealand		244	8	252	EF092962–EF092969*
	South Africa		68	21	89	AY821622–AY821634^
	Argentina		10	19	29	EF092956–EF092961*
						AY821611–AY821621^
	Peru		0	118	118	AF113492–AF113496@
						AY821573–AY821610^
		**TOTAL**	322	166	**488**	
	
		Actin^a^				
	New Zealand		48	0	48	
	South Africa		44	0	44	
	Argentina		14	0	14	
	Peru		0	3^b^	3^b^	AF140832–AF140834&
		**TOTAL**	106	3	**109**	

*L. obliquidens*		Cytb	3	6	9	EF093036–EF093041*
		D-loop	2	8	10	AF113490–AF113491@
						EF092950–EF092955*
		Actin	0	6	6	AF140826–AF140831&
		**TOTAL**	5	20	**25**	

n = number of individuals except where noted. *Harlin-Cognato and Honeycutt [10]; ^#^Cassens et al. [8]; ^^^Cassens et al. [14]; ^&^Hare et al. [12]; ^@^Cipriano [13]; ^a^n = number of gametes; ^b^n = number of individuals, phase not known.

Sequenced fragments were edited and compiled with Sequencher v. 4.1 (Gene Codes Corporation, Ann Arbor, Michigan). A consensus of sense and antisense strands for each individual sequence and data partition were compiled and exported to MacClade vs. 4.05 [26]. Amino acid translations of the open reading frames of cyt *b *were examined for stop codons to verify sequence orthology. Neither actin nor cyt *b *contained any insertions or deletions. The control region had a single indel that was accommodated in the alignment with a minor alignment adjustment. Actin sequences were subjected to a BLAST search to verify sequence orthology. In all BLAST searches, amplicons retrieved sequences from other delphinid or mammalian taxa as the closest match, providing evidence for successful amplification of the target locus.

Heterozygous nucleotide positions of the actin intron were determined objectively with the program Mutation Surveyor (SoftGenetics, State College, Pennsylvania). This software identifies with 99% accuracy heterozygous nucleotide positions, by employing an algorithm that incorporates measures of peak height, peak intensity, and background noise from ABI electrophereograms. Putative heterozygous positions identified with Mutation Surveyor were cross-verified by examination of raw electrophereogram data in Sequencher. Only cross-verified sites were used in subsequent determination of allelic variation at the locus. Allele and genotype frequencies of actin were determined statistically with the program PHASE version 2.1 [27,28]. Three independent chains of 1000 interactions each were performed with other parameters as default. Only those alleles and genotypes resolved with > 90% posterior probabilities were used in subsequent analyses.

### Diversity indices and models of evolution

Arlequin version 2.00 [29] was used to estimate haplotype diversity (i.e., gene diversity) (*h*) and nucleotide diversity (π) [30]. Standard error of these estimates was determined from a null distribution generated from 10000 random permutations of the data. DnaSP version 4.0 [31] was used to determine the number of polymorphic sites (S) from aligned sequence data.

Modeltest version 3.06 [32] was used to test 54 nested hypotheses of nucleotide substitution for each data partition. The Hasegawa, Kishino, and Yano [33] (HKY85) model with a correction for rate variation among sites (α = 0.66) and a proportion of invariant sites (I = 0.39) was selected as the best fit model for the mtDNA control region. For the cyt *b *gene, the model selected was HKY85 with a correction for rate variation among sites (α = 0.003). The actin intron also had HKY85 as the best-fit model, but with equal rates among sites. These best-fit models were used in all analyses that employed a model of evolution.

### Prediction 1: Peruvian origin

A Bayesian analysis of *cyt b *sequences from *L. obliquidens *and *L. obscurus *(Table 4), and *Lissodelphis borealis *(GenBank Accessions EF093025–EF093028) as an outgroup, was performed in Mr. Bayes version 3.1 [34]. The purpose of this analysis was to: (1) determine the geographical population at the root of the dusky dolphin origin, (2) provide support for the monophyly of Peruvian population haplotypes, and (3) provide a topology for subsequent estimation of divergence dates. The Bayesian Metropolis-coupled Markov Chain Monte Carlo analysis consisted of two independent searches of 25 × 10^6 ^steps (1 cold, 3 heated chains to insure adequate mixing) with the best-fit model of substitution for cyt *b *determined *a priori *with Modeltest. The program Tracer version 1.1.1 [35] was used to determine convergence and the burn-in point of chains. Trees prior to convergence were eliminated and a majority-rule consensus tree of the remaining topologies was constructed in PAUP* version 4.0b10 [36].

### Prediction 2: Linear dispersal route

We tested the hypothesis of a unidirectional, linear dispersal route by examining the geographic partitioning of genetic variation. The method of Templeton et al. [37] as implemented in the program TCS version 1.18 [38] was used to construct separate statistical parsimony networks for each of the three data partitions. The statistical parsimony method was chosen because of its ability to infer missing intermediates within the network, which is more appropriate for examining intra-specific relationships than standard phylogenetic methods that assume bifurcating lineages [39,40]. Furthermore, reticulations, or alternative (homoplasious) parsimonious connections, are permitted with this method, thus allowing one to test hypotheses regarding the complex historical processes that shape populations. The final network gives a visual representation of the number of mutations separating any two haplotypes (or alleles). Mitochondrial DNA haplotypes were joined to the network within 95% parsimony confidence limits [38]. Because of its slower rate of coalescence, parsimony confidence limits were expanded to 90% for actin alleles. Actin alleles of *L. obliquidens *were included in the statistical parsimony network to examine the pattern of allelic diversity at the base of the *L. obliquidens/L. obscurus *divergence. Reticulations in the actin parsimony network were observed and attributed either to lack of lineage coalescence, homoplasious substitutions, or recombination among sites. Of these, recombination is the most problematic for inferring evolutionary history from nuclear loci. Therefore, the likelihood of recombination among alleles was evaluated with the program Lamarc version 1.2.2 [41].

An analysis of molecular variance (AMOVA) [42] and F-statistics [43] were calculated with Arlequin, version 3.1 [29] to determine amount of genetic variation within and among geographic regions for cyt *b*, control region, and actin data. The significance of these statistics was determined by comparison of observed values to those derived from 10000 random permutations of the data. The "case-control" option in PHASE 2.1 was used to group actin alleles by geographic region and to subsequently test the null hypothesis of random association of alleles among localities. Test for Hardy-Weinberg equilibrium of actin genotypes was performed in Arlequin, version 3.1.

The program MIGRATE [44,45] was used to examine the pattern of dispersal (migration) between geographic regions. Although cyt b and d-loop are linked, the rate of evolution of each data partition is different with d-loop being the fastest evolving region of the mammalian mitochondrion. Therefore, the cyt b and d-loop data were analyzed independently in order to examine dispersal events over different relative time scales, under the assumption that the d-loop will provide evidence for the most contemporary dispersal/migration events. Each data partition was subjected to a maximum likelihood MCMC search strategy with initial theta values generated from F_st _estimates, and evolutionary model parameters determined from MODELTEST. Each search was comprised of a combination of 10 short and 3 long chains with an adaptive heating scheme of 4 chains (temperatures 1.0, 1.2, 1.5, 3.0) and a swap interval of 1 in order to insure adequate mixing among chains. Ten short chains of 200,000 steps were run with a sampling increment of 20, for a total of 10,000 sampled trees per chain. A total of 3 long chains were run for 20,000,000 steps, with a sampling increment of 20 for a total of 100,000 trees sampled.

### Prediction 3: Dates of divergence

We estimated dates of divergence between *L. obliquidens*/*L. obscurus *lineages and the isolation of the *L. obscurus *Peruvian population from other geographical regions to determine if historical changes in sea surface temperature, primary productivity, current systems, and the phylogeography of the dusky dolphin are correlated. The Bayesian cyt *b *phylogeny with branch lengths was subsequently used in RHINO version 1.2 [46] to estimate via maximum likelihood the date of divergence and 95% confidence intervals. A previous study of *Lagenorhynchus *cyt *b *[12] suggests the evolution of this locus is clock-like; therefore dates of divergence were calculated under the assumption of a molecular clock with substitution model parameters predetermined by Modeltest. Two calibration points were used, both strongly corroborated from the fossil record and molecular divergence estimates [47]: (1) Phocoenidae/Monodontidae, 13.0 my; (2) Phocoenida/Platanistidae, 28 myr.

### Comparative phylogeography of anchovy

Published cyt *b *sequences (n = 58) for Old World (*Engraulis capensis*, *E. japonicus*, *E. encrasicolus*) and New World (*E. mordax*, *E. ringens*, *E. anchoita*) anchovy species were assembled from GenBank (Accessions AY923766–AY923823) [48]. A Bayesian analysis was performed on a matrix of 465 base pairs, and included *Gonorhynchus greyi *(NC004702), *Chanos chanos *(NC004693), *Sardiops sagax *(AF472586), and *S. caeruleus *(AF472585) to root the phylogeny. The Bayesian Metropolis-coupled Markov Chain Monte Carlo analysis consisted of two independent searches of 2.0 × 10^6 ^steps (1 cold, 3 heated chains to insure adequate mixing) with a model of substitution referenced in Grant (reference). In order to compare dates of divergence of anchovy species and the dusky dolphin populations, the resulting topology and the program RHINO 1.2 [46] were used to estimate times of divergence of Northern Hemisphere *E. mordax *from Southern Hemisphere *E. ringens*/*E. anchoita *clade, and between western (*E. ringens*) and eastern (*E. anchoita*) South America. The oldest European anchovy fossil is 10 million years old [49], thus this date was used as a calibration point. In addition, a previously published cyt *b *haplotype network [50] was compared to that of the dusky dolphin in order to assess similarities in the geographic distribution of genetic variation. This network, plus additional, previously published phylogeographic inferences for anchovy (i.e., [6,50]) were summarized and compared to the phylogeographic history of the dusky dolphin.

## Authors' contributions

ADHC designed the study, collected tissue samples, gathered molecular data, performed data analyses, and authored the manuscript. TMM assisted with tissue sample collection and reviewed drafts of the manuscript. BW assisted with project design, support, and management, and reviewed drafts of the manuscript. RLH provided guidance for molecular data collection and analysis, assisted with project design, and helped to draft the manuscript.
